# Diurnal expression of MRP4 in bone marrow cells underlies the dosing-time dependent changes in the oxaliplatin-induced myelotoxicity

**DOI:** 10.1038/s41598-020-70321-6

**Published:** 2020-08-10

**Authors:** Mizuki Kato, Yuya Tsurudome, Takumi Kanemitsu, Sai Yasukochi, Yuki Kanado, Takashi Ogino, Naoya Matsunaga, Satoru Koyanagi, Shigehiro Ohdo

**Affiliations:** 1 Department of Pharmaceutics, Faculty of Pharmaceutical Sciences, Kyushu University, 3-1-1 Maidashi Higashi-ku, Fukuoka, 812-8582 Japan; 2 Department of Glocal Healthcare Science, Faculty of Pharmaceutical Sciences, Kyushu University, Fukuoka, Japan

**Keywords:** Adverse effects, Drug therapy

## Abstract

The expression and function of some xenobiotic transporters varies according to the time of day, causing the dosing time-dependent changes in drug disposition and toxicity. Multidrug resistance-associated protein-4 (MRP4), an ATP­binding cassette (ABC) efflux transporter encoded by the *Abcc4* gene, is highly expressed in bone marrow cells (BMCs) and protects them against xenobiotics, including chemotherapeutic drugs. In this study, we demonstrated that MRP4 was responsible for the extrusion of oxaliplatin (L-OHP), a platinum (Pt)-based chemotherapeutic drug, from BMCs of mice, and that the efflux transporter expression exhibited significant diurnal variation. Therefore, we investigated the relevance of the diurnal expression of MRP4 in BMCs for L-OHP-induced myelotoxicity in mice maintained under standardized light/dark cycle conditions. After intravenous injection of L-OHP, the Pt content in BMCs varied according to the injection time. Lower Pt accumulation in BMCs was detected in mice after injection of L-OHP at the mid-dark phase, during which the expression levels of MRP4 increased. Consistent with these observations, the myelotoxic effects of L-OHP were attenuated when mice were injected with L-OHP during the dark phase. This dosing schedule also alleviated the L-OHP-induced reduction of the peripheral white blood cell count. The present results suggest that the myelotoxicity of L-OHP is attenuated by optimizing the dosing schedule. Diurnal expression of MRP4 in BMCs is associated with the dosing time-dependent changes in L-OHP-induced myelotoxicity.

## Introduction

Daily variations in biological functions, such as gene expression and protein synthesis, are thought to be important factors affecting the efficacy and/or toxicity of drugs; a large number of drugs cannot be expected to have the same potency at different administration times^1,2^. Consequently, administration of drugs at appropriate times of day can improve the outcome of pharmacotherapy by maximizing their potency and minimizing their toxicity, whereas drug administration at an inappropriate time of day can induce severe side effects.

Dosing time-dependent differences in the drug effects are due, at least in part, to 24-h changes in drug disposition such as absorption, distribution, metabolism, and elimination^3–6^. ATP­binding cassette (ABC) transporters are widely known as energy-dependent efflux pumps that expel cytotoxic substances, including chemotherapeutic drugs. They are expressed in epithelial cells of several organs, including the brain, liver, intestine, and kidney^7^, and function as a barrier to limit intestinal absorption of many drugs and their distribution to different tissues^8^. Furthermore, several types of ABC transporters also function in the biliary, intestinal, and renal elimination of drugs^9^. The expression and function of some ABC transporters, such as P-glycoprotein (P-gp), multidrug resistance-associated protein 2 (MRP2), and breast cancer-resistant protein (BCRP), exhibit diurnal variation, resulting in dosing time-dependent differences in drug disposition^10–13^.

Myelotoxicity is a common complication when treating cancer patients using chemotherapeutic drugs. Several types of ABC transporters are detected in hematopoietic progenitor cells, suggesting that they function to protect bone marrow stem cells^14–16^. Myelotoxicity of chemotherapeutic drugs also varies depending on their administration time. Significant dosing time-dependent differences in myelosuppression were detected in experimental animals when they were administered methotrexate^17^, irinotecan^18^, doxorubicin^19^, or docetaxel^20^. Furthermore, gemcitabine-induced myelosuppression is also alleviated by administering the drug in the morning to cancer patients^21^. However, little is known about the role of xenobiotic transporters in the dosing time-dependent changes in the myelotoxicity of chemotherapeutic drugs.

Multidrug resistance-associated protein 4 (MRP4 encoded by *Abcc4* gene) is one of the ABC transporters, and is highly expressed in proximal renal tubules and bone marrow^22,23^. MRP4 recognizes a variety of compounds as substrates and transports them to the extracellular fluid^24,25^. As MRP4 knockout mice exhibit severe myelotoxicity of 6-mercaptopurine or adefovir^23,26^, this transporter may protect bone marrow cells (BMCs) against the cytotoxicity of chemotherapeutic drugs. Oxaliplatin (L-OHP) has been suggested as a substrate of MRP4^27^. L-OHP is a platinum (Pt)-based chemotherapeutic drug that is used to treat colorectal cancers. However, L-OHP can cause severe myelotoxicity, which is a dose-limiting factor of L-OHP therapy. Although the myelosuppressive effects of L-OHP are altered by its administration time^28,29^, the role of MRP4 in the dosing time-dependent changes in L-OHP-induced myelotoxicity is not well understood.

In this study, we found that the expression of MRP4 exhibited significant diurnal oscillation in BMCs of mice. This efflux transporter oscillation may underlie the dosing time-dependent differences in the accumulation of Pt in BMCs. Therefore, we investigated the relevance of diurnal expression of MRP4 in BMCs for the dosing time-dependent differences in L-OHP-induced myelotoxicity.

## Results

### Role of MRP4 in the extrusion of L-OHP from BMCs

**Figure 1:**
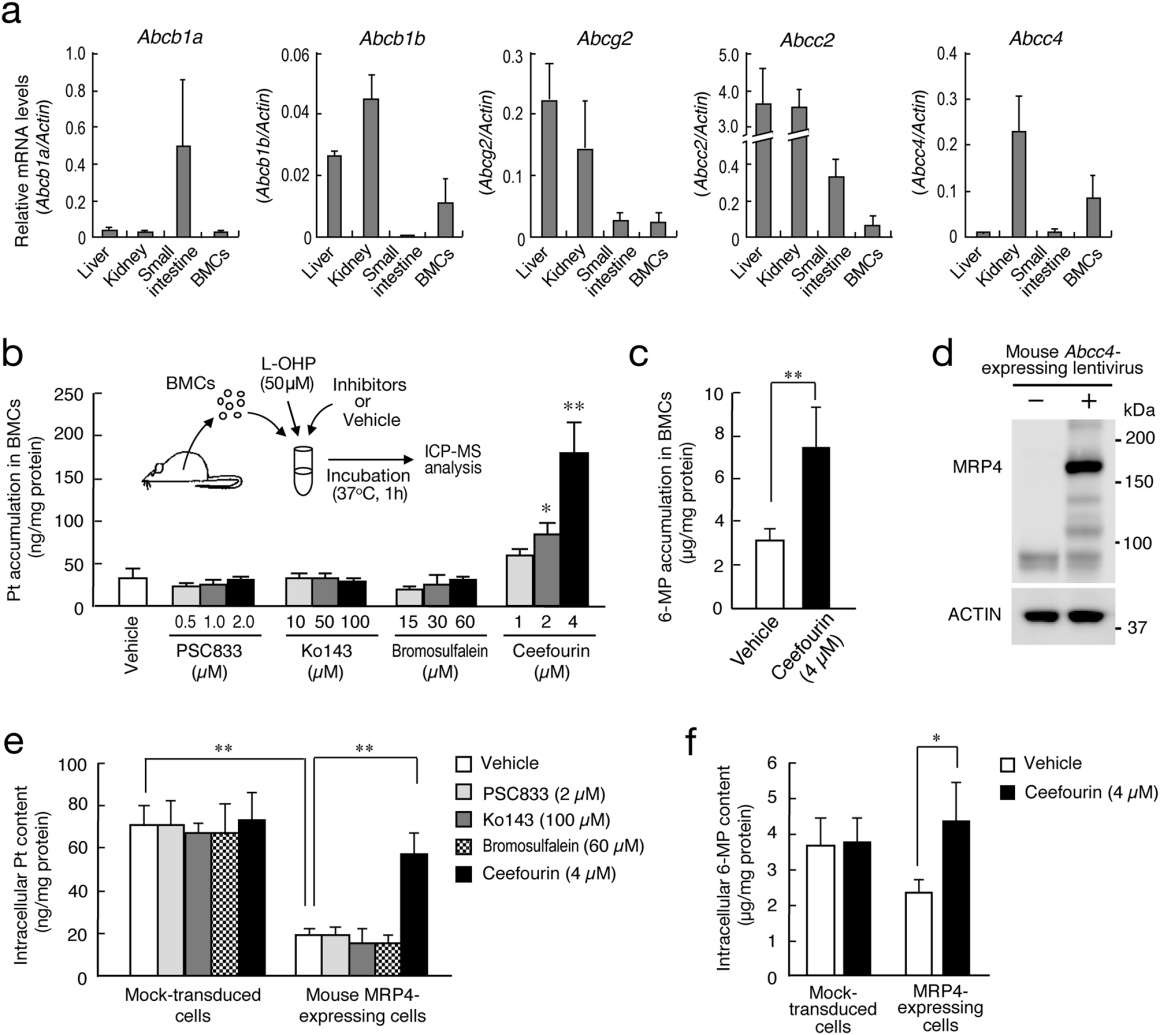
Role of MRP4 in the extrusion of L-OHP from BMCs of mice. (**a**) The mRNA expression of ABC transporters in the liver, kidney, small intestine, and BMCs of mice. The mRNA levels of ABC transporters were normalized to that of Actin. Values are the mean with S.D. (n = 3). (**b**) Effects of ABC transporter inhibitors on Pt accumulation in BMCs after treatment with L-OHP. BMCs were collected from mouse femora. Cells were incubated with 50 μM L-OHP for 1 h in the presence or absence of ABC transporter inhibitors. Values are the mean with S.D. (n = 4). **P < 0.01; *P < 0.05 significant difference compared with vehicle (0.001% DMSO) group (*F*_12,39_ = 63.654, *P* < 0.001, ANOVA with Tukey–Kramer’s post-hoc test). (**c**) Effects of ceefourin on 6-mercaptopurine (6-MP) accumulation in BMCs. BMCs were prepared as described in panel b and treated with 500 µM 6-MP for 1 h in the presence or absence of 4 µM ceefourin. Values are the mean with S.D. (n = 4). **P < 0.01 significant difference between the two groups (*t*_4_ = 4.386, *P* = 0.005, unpaired t-test, two-sided). (**d**) Construction of cells stably expressing mouse MRP4. Lentivirus expressing the mouse Abcc4 gene was transduced into NIH3T3 cells. MRP4 expression was confirmed by Western blotting. Full-size images of western blotting are presented in Supplementary Fig. 4. (**e**) Effects of ABC transporter inhibitors on Pt accumulation in mouse MRP4-expressing cells after treatment with 50 μM L-OHP for 1 h. Values are the mean with S.D. (n = 3). **P < 0.01 significant difference between the two groups (*F*_9,20_ = 28.994, *P* < 0.001, ANOVA with Tukey–Kramer’s post-hoc test). (**f**) Effects of ceefourin on 6-MP accumulation in mouse MRP4-expressing cells. Cells were treated with 50 μM L-OHP for 1 h. Values are the mean with S.D. (n = 4). **P < 0.05 significant difference between the two groups (*F*_3,12_ = 28.994, *P* = 0.018, ANOVA with Tukey–Kramer’s post-hoc test).

In addition to MRP4, the mRNA expression of major xenobiotic ABC transporters, P-gp (encoded by *Abcb1a* or *Abcb1b*), BCRP (*Abcg2*), and MRP2 (*Abcc2*), was detected in BMCs (Fig. 1a). To investigate the role of these ABC transporters in the extrusion of L-OHP from BMCs, we prepared BMCs from mouse femora, and cells were treated with 50 μM L-OHP in the presence of selective inhibitors for P-gp, BCRP, MRP2, or MRP4. After treatment of BMCs with L-OHP in the presence of the selective MRP4 inhibitor ceefourin^30^, intracellular Pt content was significantly higher than that in vehicle (0.001% DMSO)-treated cells (Fig. 1b). Treatment of BMCs with ceefourin also resulted in the accumulation of 6-mercaptopurine (6-MP), a typical substrate of MRP4 (*P* < 0.01, Fig. 1c). On the other hand, no significant accumulation of Pt was observed when BMCs were treated by L-OHP together with the P-gp inhibitor PSC833^31^, BCRP inhibitor Ko143^32^, or MRP2 inhibitor bromosulfalein^33^ (Fig. 1b).

To further investigate the role of MRP4 in the extracellular efflux of L-OHP, we prepared mouse MRP4-expressing NIH3T3 cells (Fig. 1d), which were also treated with 50 μM L-OHP in the presence or absence of PSC833, Ko143, bromosulfalein, or ceefourin. After treatment with L-OHP in the absence of ABC transporter inhibitors, intracellular Pt accumulation in MRP4-expressing cells was significantly lower than that observed in mock-transduced cells (*P* < 0.01, Fig. 1e). The low Pt accumulation in L-OHP-treated MRP4-expressing cells was abrogated by sodium orthovanadate, a Na^+^/K^+^-ATPase inhibitor (Supplementary Fig. 1), suggesting ATP-dependent extrusion of L-OHP by MRP4. After treatment of MRP4-expressing cells with L-OHP in the presence of ceefourin, intracellular Pt content was significantly increased compared with that in vehicle-treated MRP4-expressing cells (*P* < 0.01, Fig. 1e). As observed in BMCs, treatment of MRP4-expressing cells with ceefourin also caused significant accumulation of 6-MP (*P* < 0.05, Fig. 1f). On the other hand, PSC833, Ko143, and bromosulfalein had negligible effects on Pt content in L-OHP-treated MRP4-expressing cells (Fig. 1e). These results suggest that MRP4 is responsible for the extracellular efflux of L-OHP.

### Dosing time-dependent changes in L-OHP disposition in mice

**Figure 2:**
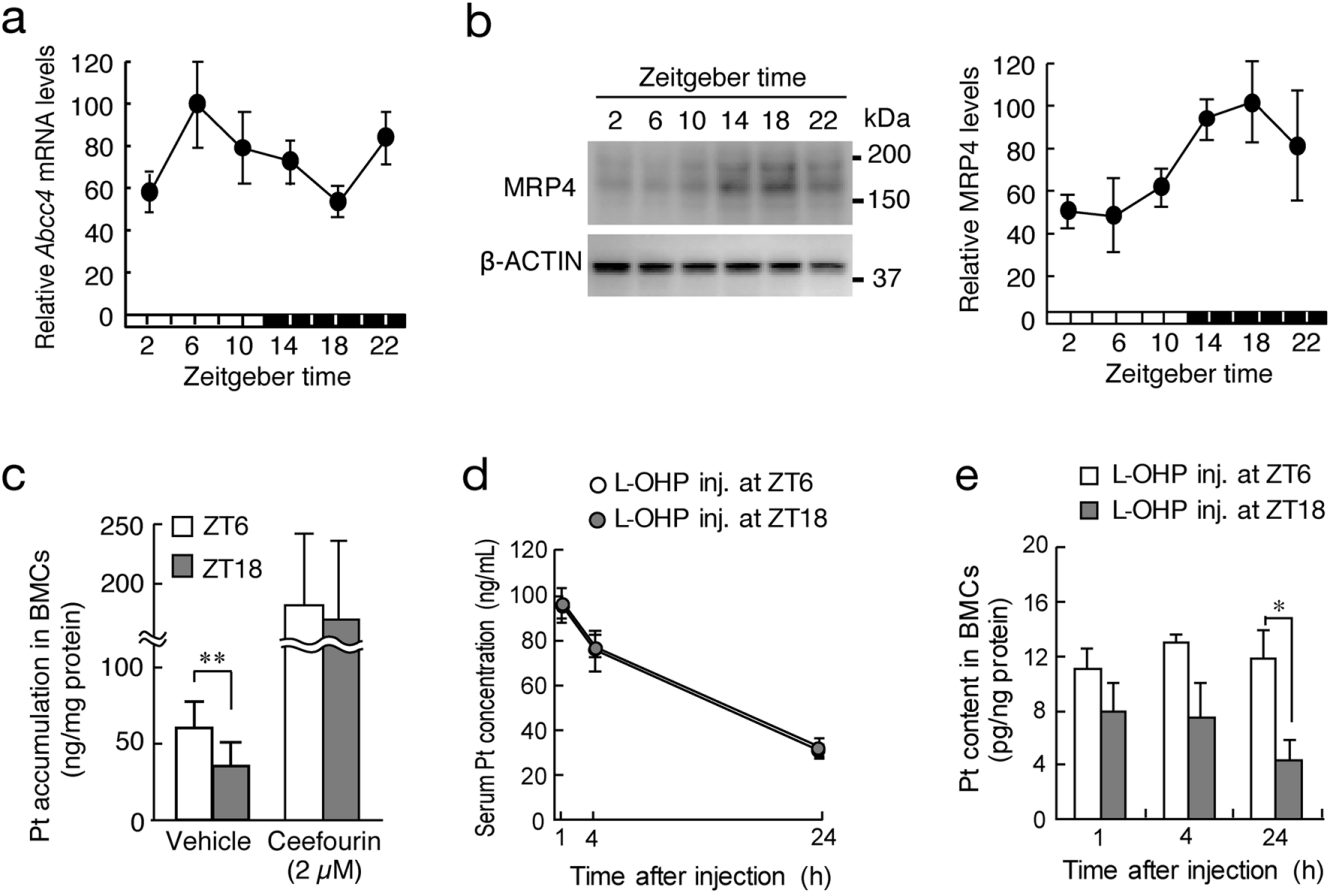
Dosing time-dependent changes in L-OHP pharmacokinetics in mice. (**a**) Temporal mRNA expression profile of *Abcc4* in BMCs of mice. The horizontal bar at the bottom indicates the light and dark cycles. Values are the mean with S.D. (n = 3). There was a significant time-dependent difference in the protein levels of MRP4 (*F*_5,12_ = 4.8254, *P* = 0.012, ANOVA). (**b**) Temporal expression profile of MRP4 in BMCs of mice. The left panel shows a representative photograph of Western blotting of MRP4. The horizontal bar at the bottom indicates the light and dark cycles. Values are the mean with S.D. (n = 3). There was a significant time-dependent difference in the protein levels of MRP4 (*F*_5,12_ = 5.626, *P* = 0.007, ANOVA). Full-size images of western blotting are presented in Supplementary Fig. 5. (**c**) Time-dependent difference in Pt accumulation in BMCs after treatment with 50 μM L-OHP in the presence or absence of 2 µM ceefourin. Values are the mean with S.D. (n = 4–6). **P < 0.01 significant difference between the two groups (*F*_3,16_ = 12.177, *P* < 0.001, ANOVA with Tukey–Kramer’s post-hoc test). (**d**) Time course of serum Pt concentration after a single dose of L-OHP (20 mg/kg, i.v.) at ZT6 or ZT18. Each point represents the mean with S.D. (n = 3). (**e**) Time course of Pt content in BMCs of mice after a single dose of L-OHP (20 mg/kg, i.v.) at ZT6 or ZT18. Values are the mean with S.D. (n = 3). *P < 0.05, significant difference between the two groups (*t*_4_ = 3.045, *P* = 0.038, unpaired t-test, two-sided).

Next, we explored the possibility that the expression and function of MRP4 vary according to the time of day. To achieve this, mice were maintained under standardized light/dark cycle conditions (Zeitgeber time 0 [ZT0], lights on; ZT12 lights off). The mRNA and membrane fractions of BMCs were prepared at six different time points. Accumulation of Pt in BMCs after L-OHP treatment was also assessed by preparing cells from mice at ZT6 and ZT18. The mRNA levels of *Abcc4* in BMCs exhibited a significant diurnal oscillation, with a peak level during the light phase (*P* < 0.05, Fig. 2a). Significant diurnal oscillation was also detected in the protein levels of MRP4 in BMCs, but the oscillation in the MRP4 protein levels was delayed by approximately 12 h relative to the *Abcc4* mRNA rhythm (*P* < 0.01, Fig. 2b). The expression of MRP4 at ZT18 was approximately twofold higher than that at ZT6. Indeed, the accumulation of Pt in BMCs prepared at ZT18 was significantly lower than that in cells prepared at ZT6 (*P* < 0.01, Fig. 2c). The time-dependent difference in the Pt accumulation in L-OHP-treated BMCs disappeared when cells were treated with the MRP4 inhibitor ceefourin. Taken together, these results suggest that diurnal expression of MRP4 induces the time-dependent change in the Pt accumulation in L-OHP-treated BMCs.

To further explore the relevance of diurnal expression of MRP4 in BMCs for the disposition of L-OHP, mice were injected with a single dose of L-OHP (17.5 mg/kg, i.v.) at ZT6 or ZT18. Although there was no significant dosing time-dependent difference in the serum Pt concentration after L-OHP injection (Fig. 2c), that in BMCs varied according to the injection time of L-OHP. A lower Pt content in BMCs was detected in mice after injection of L-OHP at ZT18 (*P* < 0.05, Fig. 2d), during which the expression level of MRP4 increased (Fig. 1b). These results suggest a close relationship between the diurnal expression of MRP4 and Pt accumulation in BMCs after injecting L-OHP.

### Dosing time-dependent changes in L-OHP-induced myelotoxicity

**Figure 3:**
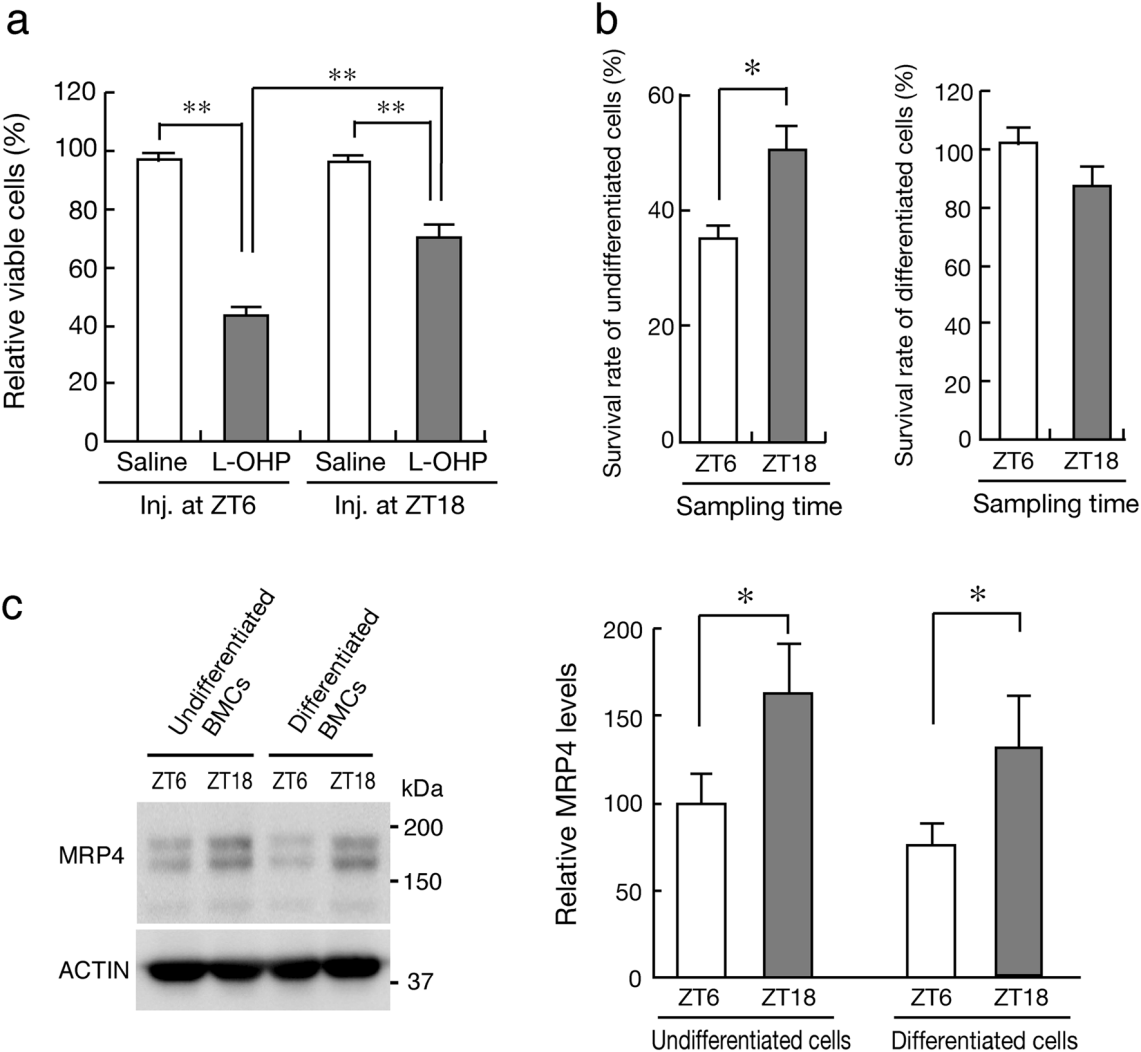
Dosing time-dependent difference in L-OHP-induced myelotoxicity in mice. (**a**) The decrease in the number of viable BMCs in mice at 72 h after injecting L-OHP (20 mg/kg, i.v.) or saline at ZT6 and ZT18. Cell viability was assessed by staining with calcein-AM on FACS. Values are the mean with S.D. (n = 4). **P < 0.01, significant difference between the two groups (*F*_3,12_ = 9.361, *P* < 0.001, ANOVA with Tukey–Kramer’s post-hoc test). (**b**) The survival rate of undifferentiated (left) and differentiated (right) BMCs after injecting L-OHP (20 mg/kg, i.v.) or saline at ZT6 and ZT18. Lineage-positive and -negative BMCs were separated by FACS as undifferentiated and differentiated cells, respectively. Values are the mean with S.D. (n = 8). *P < 0.05, significant difference between the two groups (*t*_14_ = 3.045, *P* = 0.012, unpaired t-test, two-sided). (**c**) Temporal expression profiles of MRP4 in undifferentiated and differentiated cells prepared from bone marrow of mice. Values are the mean with S.D. (n = 6). *P < 0.05, significant difference between the two groups (*F*_3,20_ = 14.892, *P* < 0.001, ANOVA with Tukey–Kramer’s post-hoc test). Full-size images of western blotting are presented in Supplementary Fig. 6.

To examine whether the dosing time-dependent difference in Pt accumulation in BMCs after the administration of L-OHP is also associated with its myelotoxicity, the number of viable BMCs was assessed after injection of L-OHP (20 mg/kg, i.v.) at ZT6 or ZT18. At 72 h after the injection of L-OHP, a significant reduction of the number of viable BMCs was detected at both dosing times (*P* < 0.01, Fig. 3a). However, the myelocytotoxic effects of L-OHP in mice injected with L-OHP at ZT18 were significantly alleviated compared with those in mice injected with the drug at ZT6 (*P* < 0.05).

Lineage is a mature blood cell marker to differentiate mature hemocytes, including T cells, B cells, monocytes, macrophages, granulocytes, and neutrophils^34^. After injecting L-OHP (20 mg/kg, i.v.) at ZT6 or ZT18, the number of lineage-positive and -negative BMCs was assessed as differentiated and undifferentiated cells, respectively. The number of viable differentiated cells was not significantly reduced after L-OHP injection at either dosing time (Fig. 3b right panel). Although a significant reduction of the number of viable undifferentiated cells was detected after L-OHP injection at both dosing times, the survival rate of undifferentiated cells after L-OHP injection at ZT18 was significantly higher than that after the drug administration at ZT6 (*P* < 0.05, Fig. 3b left panel). The expression of MRP4 slightly decreased in differentiated BMCs (Fig. 3c), but still exhibited significant diurnal variations in both differentiated and undifferentiated cells (*P* < 0.05 respectively, Fig. 3c). Therefore, the myelotoxic effects of L-OHP may be associated with the decrease in the number of undifferentiated BMCs.

### Dosing time-dependent difference in L-OHP-induced leukopenia

**Figure 4:**
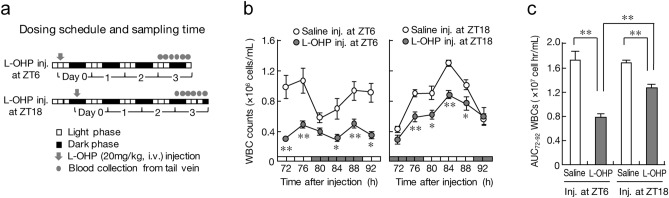
Dosing time-dependent change in L-OHP-induced leukopenia in mice. (**a**) Schematic experimental procedure for evaluating the dosing time dependency of L-OHP-induced leukopenia. Mice were injected with L-OHP (20 mg/kg, i.v.) or saline at ZT6 and ZT18. The number of peripheral WBCs was assessed from 72 to 92 h (4-h intervals) after L-OHP injection. (**b**) Temporal profiles of the number of peripheral WBCs in mice after injecting L-OHP (20 mg/kg, i.v.) or saline at ZT6 (left) or ZT18 (right). The horizontal bar at the bottom indicates the light and dark cycles. Values are the mean with S.D. (n = 6). **P < 0.01, *P < 0.05, significant difference from the saline-injected group at the corresponding time point. (**c**) Comparison of the value of AUC_72–92_ in the number of peripheral WBCs after injecting L-OHP (20 mg/kg, i.v.) or saline at ZT6 or ZT18. Values are the mean with S.D. (n = 6). **P < 0.01, significant difference between the two groups.

As hematopoietic stem cells in bone marrow proliferate and differentiate repeatedly to produce peripheral blood cells^35^, chemotherapeutic drug-induced myelotoxicity is often associated with leukopenia^36,37^. We thus examined whether L-OHP-induced leukopenia changed depending on the dosing time. The number of peripheral WBCs in mice decreased 3 days after L-OHP injection, and the peripheral WBC count varied according to the time of day in both humans and experimental rodents^17,18,38^. Therefore, mice were injected with a single dose of L-OHP (20 mg/kg, i.v.) at ZT6 or ZT18, and then the number of peripheral WBCs was assessed from 72 to 92 h (4-h interval) after L-OHP injection (Fig. 4a).

The number of WBCs in saline-treated mice exhibited significant 24-h oscillation, with a peak during the light phase (Fig. 4b). A significant reduction of the number of peripheral WBCs was observed in mice injected with L-OHP at both time points, but the L-OHP-induced reduction of the peripheral WBC count was attenuated by injection of the drug at ZT18 (Fig. 4b). Indeed, the value of the area under the curve (AUC) of the time course of the peripheral WBC count (AUC_72-92_ of WBCs) after L-OHP injection at ZT6 was markedly lower than that after the drug injection at ZT18 (Fig. 4c). This demonstrated that L-OHP-induced leukopenia also varies according to its administration time. The dosing time-dependent difference in the myelotoxic effects of L-OHP may affect the severity of leukopenia.

## Discussion

Diurnal oscillations in the activity of drug metabolism enzymes and xenobiotic transporters are often associated with the dosing time-dependent changes in drug disposition^1,2,38^. Such dosing time-dependency of drug pharmacokinetics also affects their efficacy and safety. In this study, we demonstrated that the expression of MRP4 in murine BMCs varies according to the time of day. The diurnal expression of efflux transporters was associated with the dosing time-dependent difference in L-OHP-induced myelotoxicity in mice.

In addition to MRP4, the mRNA expression of P-gp (encoded by *Abcb1a* or *Abcb1b*), BCRP (*Abcg2*), and MRP2 (*Abcc2*) was detected in BMCs of mice. However, no significant accumulation of Pt was observed in BMCs after treatment with L-OHP in the presence of inhibitors. Treatment of BMCs by L-OHP together with ceefourin resulted in a 5-times increase in Pt accumulation. Ceefourin was originally reported as a human MRP4 inhibitor^29^, but it also induced significant Pt accumulation in mouse MRP4-expressing cells after L-OHP treatment. Ceefourin may exert its inhibitory action on both human and mouse MRP4. Collectively, these findings suggest that MRP4 functions as a major efflux transporter of L-OHP in mouse BMCs.

In contrast to efflux transporters, human organic cation transporter 2 and 3 (hOCT2 and hOCT3), human novel organic cation transporter 1 and 2 (hOCTN1 and hOCTN2), and human apical multidrug and toxin extrusion1 and 2-K (hMATE1 and hMATE2-K) have been suggested to incorporate L-OHP into cells^39–41^. Although the mRNA expression of OCTN1 (encoded by *Slc22a4*), but not of OCT2 (*Slc22a2*), OCT3 (*Slc22a3*), OCTN2 (*Slc22a5*), or MATE1 (*Slc47a1*), was detected in BMCs of mice, the protein levels of OCTN1 failed to exhibit significant diurnal oscillation (Supplementary Fig. 2). In the mouse small intestine, OCTN1 is expressed in a diurnal time-dependent manner^5^. The oscillation of intestinal expression of OCTN1 is dependent on food-entrainable bile acid stimulation; however, it is unlikely that bile acid reaches bone marrow and affects the expression of OCTN1. This may explain the reason for the lack of diurnal expression of OCTN1 in BMCs. Although other xenobiotic transporters may also participate in the influx and efflux of L-OHP in BMCs, diurnal expression of MRP4 is, at least in part, associated with the dosing time-dependent difference in Pt accumulation in BMCs after treatment with L-OHP. This is also supported by the present findings that the time-dependent change in Pt accumulation in L-OHP-treated BMCs disappeared after inhibition of MRP4 activity.

Diurnal expression of some types of ABC transporters are regulated by the molecular circadian clock^10, 11,13^. Gene products of *Clock* and *Bmal1* form a heterodimer that activates the transcription of *Period* (*Per*) and *Cryptochrome* (*Cry*) genes through E-box (CACGTG) or E-box-like (CACGTT) enhancer sequences^42^. Once PER and CRY proteins have reached a critical concentration, they attenuate CLOCK/BMAL1-mediated transactivation^43,44^, thereby generating diurnal oscillation in their own transcription. CLOCK and BMAL1 also activate their target genes, but this transactivation is periodically repressed by PER and CRY, resulting in diurnal oscillation in the downstream events^45,46^. Proline- and acid-rich (PAR) basic leucine zipper (bZIP) proteins, hepatic leukemia factor (HLF), thyrotroph embryonic factor (TEF), and D-site binding protein (DBP) are examples of such output mediators^47,48^. Previous studies have demonstrated that renal expression of *Abcc4* mRNA is decreased in PAR bZIP protein-deficient mice^49^. As the circadian clock genes and clock-controlled output mediators are also expressed in BMCs of mice^50^, these molecular components may generate diurnal expression of *Abcc4* mRNA and MRP4 protein in BMCs.

The dosing time-dependency of the pharmacological effects of drugs is attributable not only to the diurnal changes in their pharmacokinetics, but also to the sensitivity of cells to drugs^1,35^. Intracellularly incorporated L-OHP forms Pt–DNA adducts and activates apoptotic signals, resulting in cell death. One possible signal of L-OHP-induced apoptosis is the blockage of RNA polymerases by Pt–DNA adducts, causing transcription cessation and cell death through p53-dependent pathways^51^. The expression of p53 in mouse BMCs exhibits diurnal oscillation, with a peak during the light phase^52^. As severe myelotoxic effects of L-OHP were observed when mice were injected with the drug during the light phase, the temporal p53 accumulation in BMCs may also be associated with dosing time-dependent changes in L-OHP-induced myelotoxicity.

WBCs are produced and derived from multipotent cells in the bone marrow known as hematopoietic stem cells. Diurnal release and accumulation of hematopoietic stem cells in the circulating blood is important for the regeneration of the stem cell niche in bone marrow^53^, resulting in diurnal changes in the number of circulating WBCs. Diurnal release of hematopoietic stem cells from bone marrow is regulated by CXCL12 whose microenvironmental expression is controlled by core components of the molecular clock through circadian noradrenaline secretion by the sympathetic nervous system. The cellular sensitivity to chemotherapeutic drugs differs between undifferentiated and differentiated hematopoietic stem cells^54^. The cytotoxic effects of L-OHP were only detected in undifferentiated BMCs, even though diurnal expression of MRP4 was noted in both undifferentiated and differentiated BMCs. Marked cytotoxicity of L-OHP in undifferentiated BMCs was observed when mice were injected with the drug during the light phase. This may also explain the dosing time-dependent difference in L-OHP-induced leukopenia.

Significant dosing time-dependent differences in myelosuppression were detected in experimental animals when they were administered irinotecan or doxorubicin^17,18^. Although the active metabolites of irinotecan SN-38 and doxorubicin have been reported as substrates of MRP4^55^, there was no significant time-dependent accumulation of these drugs in mouse BMCs (Supplementary Fig. 3). In addition, accumulation of SN-38 and doxorubicin in mouse BMCs were not affected by the MRP4 inhibitor ceefourin. The extracellular extrusion of SN-38 and doxorubicin may be caused by other xenobiotic transporters whose expression does not exhibit diurnal oscillation in mouse BMCs. On the other hand, dosing time-dependent differences in myelosuppression by SN-38 and doxorubicin may be mainly associated with diurnal changes in the sensitivity of BMCs to drugs rather than their pharmacokinetics. The activity of topoisomerase, a target enzyme of SN-38 and doxorubicin, has been reported to exhibit diurnal oscillation in mouse BMCs^18^.

In humans, leukopenia induced by L-OHP also varies with the administration time^28^. However, the underlying mechanism is not fully understood. Our present study suggests a possible mechanism of the dosing time-dependent difference in L-OHP-induced myelotoxicity and leukopenia. Although many chemotherapeutic drugs often cause severe leukopenia, they are still administered without considering the time of day. Identification of the mechanism underlying the dosing time-dependent toxicity of chemotherapeutic drugs is expected to improve pharmacotherapy for the treatment of cancer.

## Experimental procedures

### Animals and treatments

All animal experiments were conducted in accordance with the Guidelines for Animal Experiments of Kyushu University and were approved by the Institutional Animal Care and Use Committee of Kyushu University (approved protocol ID #A30-060-0). Five-week-old male ICR mice (Charles River) were housed 6 per cage in a temperature-controlled (24 ± 1 °C) room under a 12-h light/dark cycle (ZT0, lights on; ZT12 lights off) with food and water ad libitum. During the dark period, a dim red light was used to aid animal treatment.

### Preparation of mouse MRP4-expressing cells

To establish cells stably expressing mouse MRP4, full-length mouse *Abcc4* cDNA was subcloned into lentiviral vectors under the control of the EF1α promoter. Lentivirus particles were prepared by the Lentiviral High Titer Packaging Mix with pLVSIN series (Clonetech, Palo Alto, CA) with Lenti-X 293 T cell lines. NIH3T3 cells were infected with MRP4-expressing lentivirus and maintained in a medium containing 5 µg/mL of puromycin.

### Quantitative RT-PCR analysis

The mRNA levels of ABC transporter were quantified as described in our previous report^56^. Total RNA was extracted using RNAiso PLUS reagent (Takara, Osaka, Japan). cDNA was synthesized using 1 µg of RNA and the ReverTra Ace qPCR RT kit (Toyobo, Osaka, Japan). The cDNA was amplified by a real-time PCR system (Applied Biosystems, Life Technologies, Carlsbad, CA) using specific primer sets. Sequences of primers are listed in Supplementary Table 1.

### Measurement of Pt content

Cells were incubated in transport buffer (130 mM NaCl, 6.0 mM KCl, 12 mM D-glucose, 2.0 mM CaCl_2_, 1.2 mM MgCl_2_, 0.1 mM CH_3_COONa, and 6.0 mM HEPES) for 15 min. After pre-incubation, cells were incubated in transport buffer containing 50 μM L-OHP at 37 °C in the presence or absence of ABC transporter inhibitors, PSC833, Ko143, bromosulfalein, or ceefourin, at 37 °C for 1 h. After washing with ice-cold PBS for two times, the amount of Pt in cells was measured by inductively coupled plasma mass spectrometry (ICP-MS; Agilent7500c; GenTech Science Inc., Frankfurt, Germany). The intracellular Pt content was normalized by protein concentrations.

### Measurement of 6-MP content

After pre-incubation of cells with transport buffer for 15 min, cells were incubated in transport buffer containing 1 mM 6-MP at 37 °C in the presence or absence of ceefourin at 37 °C for 1 h. After washing with ice-cold PBS for two times, cells were homogenized with 1 mL of acetonitrile containing 50 µg of 6-thioguanine (internal standard) and 10 nmol of dithiothreitol. The mixture was centrifuged at 12,000 rpm for 10 min, and the organic phase was collected and added to 1.5 mL of dichloromethane. The mixture was centrifuged for 10 min. The supernatant was subjected to HPLC after evaporation to dryness. The mobile phase of phosphate buffer (pH 2.5; 10 mM)-acetonitrile (96:4, v/v) containing 0.015% dithiothreitol was eluted at 1.0 mL/min through a C18-MS-II column (4.6 × 150 mm; Nacalai Tesque, Kyoto, Japan) using an LC-20AD pump (Shimadzu Corporation, Kyoto, Japan). The separated analyte was detected at a wavelength of 323 nm using SPD-20A (284 nm; Shimadzu).

### Western blot analysis

The levels of MRP4 protein were quantified as described in our previous report^57^. BMCs prepared from mouse femora were homogenized in lysis buffer containing appropriate protease inhibitors (100 μM phenylmethanesulfonyl fluoride, 2 μg/ml of leupeptin, and 2 μg/ml of aprotinin) and then centrifugated at 4 °C for 10 min at 12,000 × g. The supernatants were denatured at 99 °C for 5 min with 1% SDS and 5% 2-mercaptethanol. Denatured samples containing 20 µg of protein were separated by SDS-PAGE and transferred to a PVDF membrane. The membranes were reacted with antibodies against MRP4 (1:5,000; ab15602; Abcam, Cambridge, UK) and β-ACTIN (1:2000; sc-1616; Santa Cruz Biotechnology, Texas, USA). Specific antigen–antibody complexes were visualized using horseradish peroxidase-conjugated secondary antibodies (1:10,000; sc-2032; Santa Cruz Biotechnology) and ImmunoStar LD (Wako chemicals).

### Measurement of the number of white blood cells in peripheral blood

To investigate the dosing time-dependent differences in L-OHP-induced myelotoxicity, mice were injected with a single dose of 20 mg/kg (i.v.) of L-OHP at ZT6 or ZT18. Peripheral blood was collected from the mouse tail vein at ZT6 and ZT18, and 20-µL blood samples were incubated with 1 mL of hemolytic buffer (0.75% NH_4_Cl and 17 mM Tris, pH = 7.65, 37 °C) for 15 min. After centrifugation, white blood cells (WBCs) were collected and fixed with 4% paraformaldehyde for 10 min. After nuclear staining with 4′,6-diamidino-2-phenylindole (Dojindo Laboratories, Kumamoto, Japan), the numbers of WBCs were measured using an automatic cell counting instrument (Thermo Fisher Scientific, San Jose, CA).

### Pharmacokinetic analysis of L-OHP

Mice were injected with a single dose of L-OHP (17.5 mg/kg, i.v.) at ZT6 and ZT18. Serum and BMCs were collected at 1, 4, and 24 h after L-OHP administration. Pt concentrations in serum and BMCs were measured by ICP-MS as described above.

### Measurement of BMC viability

BMCs were collected from mouse femora at 72 h after the administration of L-OHP. After hemolysis, as described above, cell suspensions were prepared in 1 mL of PBS containing 1% bovine serum albumin (BSA). The number of cells was counted using an automatic cell counting instrument. Thereafter, the number of cells was adjusted to 1.0 × 10^6^/mL and they were treated with 50 μM calcein-AM (Live/Dead Cell Staining Kit II; PromoKine, Heidelberg, Germany) for 45 min at room temperature under light shielding. Calcein-stained cells were counted using a flow cytometer (FACS; BD Biosciences).

### Measurement of the number of differentiated and undifferentiated BMCs

BMCs were collected from mouse femora and treated with hemolytic agent. Cells were suspended in 1 mL of 1% BSA/PBS solution and the number of cells was adjusted to 1.0 × 10^6^/500 μL. After adding 1 μL of Lineage Cell Detection Cocktail-Biotin solution (Miltenyi Biotec, Bergisch Gladbach, Germany), the cells were incubated at 4 °C for 10 min, and then further incubated for 10 min at 4 °C in the presence of fluorescein avidin (Vector Laboratories, CA) under light shielding. Using FACS, lineage-positive and -negative cells were counted as differentiated and undifferentiated cells, respectively.

### Statistical analysis

The significance of differences among groups was analyzed by ANOVA, followed by the Tukey–Kramer post-hoc test. The unpaired t-test was used for the comparison of data between two groups. Equal variances were not formally tested. A 5% level of probability was considered to be significant.

## Supplementary information

Supplementary Information.

## Data Availability

All data supporting the results of the present study are included in the article, either in the main figures or supplementary information files.
