# Supplementary material for: Diurnal expression of MRP4 in bone marrow cells underlies the dosing-time dependent changes in the oxaliplatin-induced myelotoxicity
Source: Sci Rep. 2020 Aug 10;10:13484. doi: 10.1038/s41598-020-70321-6 (PMC7417537; doi:10.1038/s41598-020-70321-6)
Supplement: Supplementary file 1 — Supplementary Information. [file 41598_2020_70321_MOESM1_ESM.pdf]

Supplementary information

Diurnal expression of MRP4 in bone marrow cells underlies the dosing-time dependent changes in the oxaliplatin-induced myelotoxicity

Mizuki Kato[1], Yuya Tsurudome[1], Takumi Kanemitsu[1], Sai Yasukochi[1], Yuki Kanado[1], Takashi Ogino[1], Naoya Matsunaga[1,2], Satoru Koyanagi[1,2], Shigehiro Ohdo[1]

1. Department of Pharmaceutics, Faculty of Pharmaceutical Sciences, Kyushu University, Fukuoka, Japan
2. Department of Glocal Healthcare Science, Faculty of Pharmaceutical Sciences, Kyushu University, Fukuoka, Japan

Correspondence should be addressed to S.O. (ohdo@phar.kyushu-u.ac.jp)

Contents

- Supplementary methods
- Supplementary Table 1 Primer sets for PCR analysis of ABC transporter gene expression.
- Supplementary Table 2 Primer sets for PCR analysis of SLC transporter gene expression.
- Supplementary Figure 1 Effect of Na+/K+ ATPase inhibitor Na_3VO_4 on the extrusion of L-OHP from MRP4-expressing cells.
- Supplementary Figure 2 The mRNA expression of SLC transporters in liver, kidney, small intestine, and BMCs of mice.
- Supplementary Figure 3 Accumulation of SN-38 and doxorubicin in BMCs.
- Supplementary Figure 4 Unedited full blots of Figure 1d.
- Supplementary Figure 5 Unedited full blots of Figure 2b.
- Supplementary Figure 6 Unedited full blots of Figure 3c.

Supplementary methods

Quantitative RT-PCR analysis. The mRNA levels of ABC transporters were quantified as described in our previous report [1]. Total RNA was extracted using RNAiso PLUS reagent (Takara, Osaka, Japan). cDNA was synthesized using 1 µg of RNA and the ReverTra Ace qPCR RT kit (Toyobo, Osaka, Japan). The cDNA was amplified by a real-time PCR system (Applied Biosystems, Life Technologies, Carlsbad, CA) using specific primer sets. Sequences of primers are listed in **Supplementary Table 2**.

Western blot analysis. The levels of MRP4 protein were quantified as described in our previous report [2]. BMCs prepared from mouse femora were homogenized in lysis buffer containing appropriate protease inhibitors (100 µM phenylmethanesulfonyl fluoride, 2 µg/ml of leupeptin, and 2 of µg/ml aprotinin) and then centrifugated at 4°C for 10 minutes at 12,000 × g. The supernatants were denatured at 99°C for 5 minutes with 1% SDS and 5% 2-mercaptethanol. Denatured samples containing 20 µg of protein were separated by SDS-PAGE and transferred to a PVDF membrane. The membranes were reacted with antibodies against OCTN1 (OCTN11-A; 1:3000; Alpha Diagnostic International, San Antonio, TX) and β-ACTIN (1:2000; sc-1616; Santa Cruz Biotechnology, Texas, USA). Specific antigen-antibody complexes were visualized using horseradish peroxidase-conjugated secondary antibodies (1:10000; sc-2032; Santa Cruz Biotechnology) and ImmunoStar LD (Wako chemicals).

Measurement of SN-38 content. After pre-incubation of cells with transport buffer for 15 min, cells were incubated in transport buffer containing 500 µM SN-38 at 37°C in the presence or absence of ceefourin at 37°C for 1 h. After washing with ice-cold PBS twice, cells were homogenized with 1 mL of methanol containing 0.1 mM diisopropyl fluorophosphate and 5 µg of camptothecin (internal standard), and then centrifuged at 3,000 rpm for 10 min to deproteinate the samples. The supernatant was evaporated, and the residue was dissolved in 200 µL of a solution containing tetrahydrofuran/50 mM KH_2PO_4 and 5 mM heptanesulfonate (25:75, v/v), pH 2.0. The insoluble substance was removed by centrifugation at 10,000 rpm for 5 min. The solution (20 µL) was injected into the HPLC system, which comprised an LC-20AD pump (Shimadzu Corporation, Kyoto, Japan), RF-20A xs fluorometric detector (Shimadzu), and C18-MS-II column (4.6×150 mm; Nacalai Tesque, Kyoto, Japan). The mobile phases consisted of tetrahydrofuran/50 mM KH_2PO_4 and 5 mM heptanesulfonate (32:68 v/v, pH 4.0). The flow rate was 1.0 mL/min. The fluorospectromonitor was set at an excitation wavelength of 380 nm and an emission wavelength of 550 nm.

Measurement of doxorubicin content. After pre-incubation of cells with transport buffer for 15 min, they were incubated in transport buffer containing 500 μM doxorubicin at 37°C in the presence or absence of ceefourin at 37°C for 1 h. After washing with ice-cold PBS twice, cells were homogenized with 1 mL of acetonitrile containing 1 μg of daunorubicin (internal standard), and then centrifuged at 3,000 rpm for 10 min to deproteinate the samples. The supernatant was filtered through 0.45-μm filters. The solution (20 μL) was injected into the HPLC system, which comprised an LC-20AD pump (Shimadzu Corporation, Kyoto, Japan), RF-20A xs fluorometric detector (Shimadzu), and C18-MS-II column (4.6×150 mm; Nacalai Tesque, Kyoto, Japan). The mobile phase of water:acetonitrile (30:70, pH 3.0, adjusted with phosphoric acid) was delivered at a flow rate of 1.0 mL/min. The mobile phase was delivered by the gradient method using acetonitrile at 15% at 0 min, 25% at 5 min, 40% at 10 min, and 70% at 15 min. The fluorospectromonitor was set at an excitation wavelength of 470 nm and an emission wavelength of 555 nm.

References

1. Hashikawa, K.I., Katamune, C., Kusunose, N., Matsunaga, N., Koyanagi, S., & Ohdo, S. Dysfunction of the circadian transcriptional factor CLOCK in mice resists chemical carcinogen-induced tumorigenesis. *Sci. Rep.* **7**, 9995 (2017).
2. Tsurudome, Y., Koyanagi, S., Kanemitsu, T., Katamune, C., Oda, M., Kanado, Y., Kato, M., Morita, A., Tahara, Y., Matsunagam N., Shibata, S., & Ohdo, S. Circadian clock component PERIOD2 regulates diurnal expression of Na+/H+ exchanger regulatory factor-1 and its scaffolding function. *Sci. Rep.* **8**, 9072 (2018).

Supplementary Table 1 Primer sets for PCR analysis of ABC transporter gene expression

Gene		Primers
**Mouse *Abcb1a***		
	Forward	5’-GTCCCAACTGGGATATTGTACA-3’
	Reverse	5’-AGGTGCCCATGTCTGAGTAA-3’
**Mouse *Abcb1b***		
	Forward	5’-CGCCAGGCTTGCTGTAGTTAC-3’
	Reverse	5’-TGACTCCTGTCCCGAGGTTT-3’
**Mouse *Abcg2***		
	Forward	5’-AGCTCCGATGGATTGCCAG-3’
	Reverse	5’-GAGGGTTCCCGAGCAAGTTT-3’
**Mouse *Abcc2***		
	Forward	5’-ACTGGACAAGCCACAATTCC-3’
	Reverse	5’-CTGCAGGAGTGCTCGTATCA-3’
**Mouse *Abcc4***		
	Forward	5’-CCTGGAATCCACAACACGGA-3’
	Reverse	5’-TTTGTAAGCCCGGATGGTCC-3’
**Mouse *β-Actin***		
	Forward	5’-GGCTGTATTCCCCTTCCATCG-3’
	Reverse	5’-CCAGTTGGTAACAATGCCATGT-3’

Supplementary Table 2 Primer sets for PCR analysis of SLC transporter gene expression

Gene		Primers
**Mouse *Slc22a2***		
	Forward	5'-AGCCTGCCTAGCTTCGGTTT-3'
	Reverse	5'-TGCCCATTCTACCCAAGCA-3'
**Mouse *Slc22a3***		
	Forward	5'-ACTTAATATCCTGTTTCGGC-3'
	Reverse	5'-CTTTCCAAACACTCCTTGTA-3'
**Mouse *Slc22a4***		
	Forward	5'-CCTGTTCTGTGTTCCCCTGT-3'
	Reverse	5'-GGTTATGGTGGCAATGTTCC-3'
**Mouse *Slc22a5***		
	Forward	5'-AACTCACGAGCCTTGCACGCAGA-3'
	Reverse	5'-TCACCTCGTCGTAGTCCCGCA-3'
**Mouse *Slc47a1***		
	Forward	5'-AGGCCAAGAAGTCCTCAGCTATT-3'
	Reverse	5'-ACGCAGAAGGTCACAGCAAA-3'
**Mouse *β-Actin***		
	Forward	5'-GGCTGTATTCCCCTTCCATCG-3'
	Reverse	5'-CCAGTTGGTAACAATGCCATGT-3'

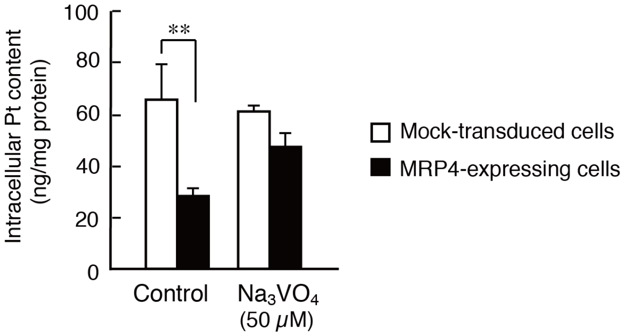

Supplementary Figure 1 Effects of the Na^{+}/K^{+} ATPase inhibitor Na_3VO_4 on the extrusion of L-OHP from MRP4-expressing cells. Mock-transduced and mouse MRP4-expressing cells were treated with 50 μM L-OHP in the presence or absence of 50 μM Na_3VO_4 for 1 h. Intracellular Pt content was assessed by ICP-MS. Values are the mean with S.D. (n = 3). **; $P < 0.01$ significant difference between the two groups ($F_{3,8} = 17.765$, $P < 0.001$, ANOVA with Tukey–Kramer's post-hoc test).

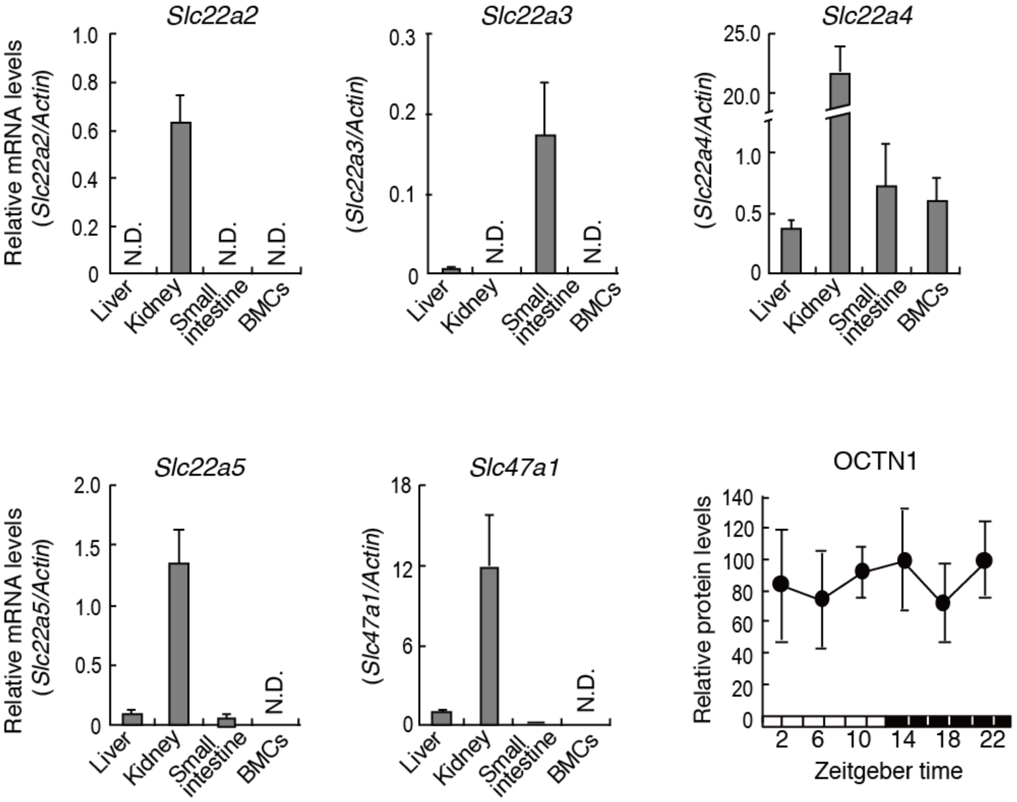

Supplementary Figure 2 mRNA expression of SLC transporters in the liver, kidney, small intestine, and BMCs of mice. The mRNA levels of SLC transporters were normalized to Actin. N.D. means not detected. The lower right panel shows the temporal expression profile of OCTN1 protein in BMCs of mice. The horizontal bar at the bottom indicates the light and dark cycles. Values are the mean with S.D. (n = 3).

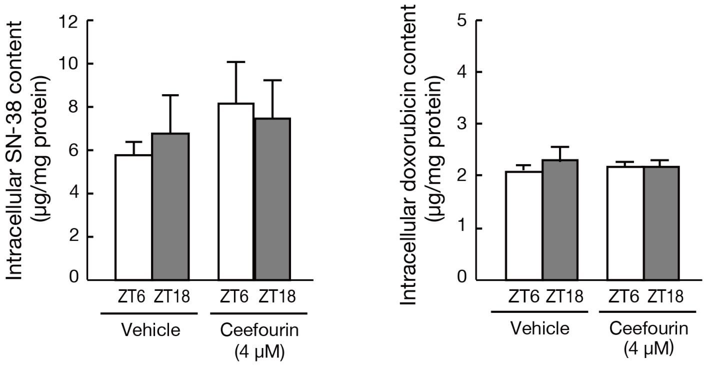

Supplementary Figure 3 Accumulation of SN-38 and doxorubicin in BMCs. BMCs were collected from mouse femora at ZT6 and ZT18. Cells were incubated with 500 μM SN-38 or 100 μM doxorubicin for 1 h in the presence or absence of 4 μM ceefourin. Values are the mean with S.D. (n = 3).

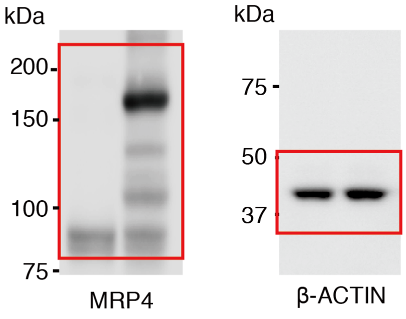

Supplementary Figure 4 Unedited full blots of Figure 1d

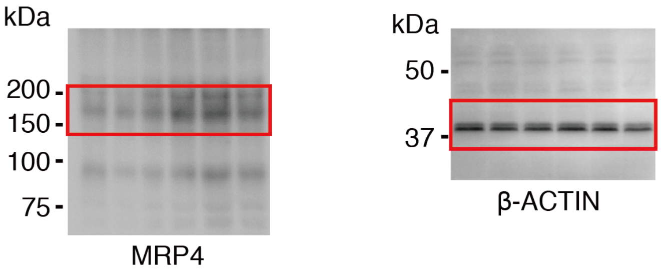

Supplementary Figure 5 Unedited full blots of Figure 2b

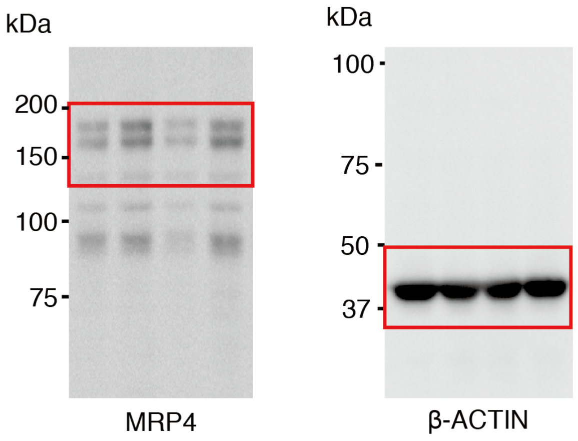

Supplementary Figure 6 Unedited full blots of Figure 3c
